# Oscillatory shear stress promotes vein graft intimal hyperplasia *via* NADPH oxidase-related pathways

**DOI:** 10.3389/fsurg.2023.1073557

**Published:** 2023-02-13

**Authors:** Guoqing Yao, Huanhuan Li, Xiangyi Zuo, Chunkai Wang, Yelei Xiao, Yu Zhao, Xuehu Wang

**Affiliations:** ^1^ Department of Vascular Surgery, The First Affiliated Hospital of Chongqing Medical University, Chongqing, China; ^2^ Department of Emergency, Chongqing University Three Gorges Hospital, Chongqing, China

**Keywords:** oscillating shear stress, endothelial proliferation, NOX, reactive oxygen species, revascularization

## Abstract

**Background:**

Uncontrolled intimal hyperplasia (IH) after autologous saphenous vein grafting triggers a high restenosis rate; however, its association with the activation of NADPH oxidase (NOX)-related pathways is unclear. Here, we investigated the effects and mechanism of oscillatory shear stress (OSS) on grafted vein IH.

**Methods:**

Thirty male New Zealand rabbits were randomly divided into control, high-OSS (HOSS), and low-OSS (LOSS) groups, and the vein grafts were harvested after 4 weeks. Hematoxylin and eosin staining and Masson staining assays were used to observe morphological and structural changes. Immunohistochemical staining was used to detect *α*-SMA, PCNA, MMP-2, and MMP-9 expression. Immunofluorescence staining was used to observe reactive oxygen species (ROS) production in the tissues. Western blotting was used to determine the expression levels of pathway-related proteins (NOX1, NOX2, AKT, *p*-AKT, and BIRC5), PCNA, BCL-2, BAX, and caspase-3/cleaved caspase-3 in tissues.

**Results:**

Blood flow velocity was lower in the LOSS group than in the HOSS group, while vessel diameter did not change significantly. Shear rate was elevated in both HOSS and LOSS groups but was higher in the HOSS group. Additionally, vessel diameter increased with time in the HOSS and LOSS groups, whereas flow velocity did not. Intimal hyperplasia was significantly lower in the LOSS group than in the HOSS group. IH was dominated by smooth muscle fibers in the grafted veins and collagen fibers in the media. OSS restriction significantly reduced the *α*-SMA, PCNA, MMP-2, and MMP-9 levels. Moreover, ROS production and the expression of NOX1, NOX2, *p*-AKT, BIRC5, PCNA, BCL-2, BAX, and cleaved caspase-3 were phase-reduced in LOSS compared to the levels in the HOSS group. Total AKT was not differentially expressed among the three groups.

**Conclusion:**

OSS promotes the proliferation, migration, and survival of subendothelial vascular smooth muscle cells in grafted veins, which may be related to the regulation of downstream *p*-AKT/BIRC5 levels through the increased production of ROS by NOX. Drugs inhibiting this pathway might be used to prolong vein graft survival time.

## Introduction

Coronary artery bypass grafting and peripheral revascularization are the most common treatments for arterial occlusion, with autologous saphenous vein grafts being the most popular grafts (1). After venous grafting, the graft undergoes a process of “arterialization,” which causes complex changes by hemodynamic factors in the lumen of the grafted vein owing to different graft characteristics (2–4). These include proliferation of subendothelial vascular smooth muscle cells (VSMCs) and deposition of the extracellular matrix, both of which accelerate intimal hyperplasia (IH) in the grafted vein. Uncontrolled IH leads to vein graft failure and serious clinical complications.

There is growing evidence that hemodynamic changes initiate the development of many vascular diseases (5, 6). In the pathogenesis of vascular disease, vascular endothelial cells (VECs) can convert mechanical stimuli, such as wall shear stress (WSS), into biological signals that promote the proliferation, migration, and survival of VSMCs. Such pathological events ultimately lead to IH and vascular stenosis. WSS is a stress exerted on the inner wall of the lumen by the passage of liquid. The WSS of blood flow is a stress composed of magnitude and vector, while the oscillatory shear stress (OSS) is a regular stress that is inconsistent with the overall direction of blood flow (7). In the physiological state, a certain degree of high flow shear stress has a protective effect on VECs (8). However, recent studies have shown that fluctuating WSS, particularly oscillatory shear stress (OSS), can significantly accelerate the development of IH (9–11). Furthermore, OSS-derived vein graft IH involves multiple signaling pathways, which activate oxidative stress factors and cause an imbalance between endogenous oxidants and antioxidants in VSMCs.

Oxidative stress results from the sustained production of reactive oxygen species (ROS), most of which are produced by mitochondrial enzymes, such as nicotinamide adenine dinucleotide phosphate (NADPH) oxidase (NOX) and xanthine oxidase (12). The NOX family consists of seven members, including NOX 1–5, dual-function oxidase 1 (DUOX1), and DUOX2. NOX1, 2, 4, and 5 are mainly expressed in VSMCs and VECs (13). NOX1 and NOX2 are generally considered promote the proliferation and migration of VSMCs under pathological conditions (14, 15). NOX4 is essential for the maintenance of the differentiation of VSMCs, regulating troponin and serum response factors to support the expression of differentiation genes under non-stimulatory conditions, while antagonizing the proliferative effect of NOX1 (16). Rabbit NOX5 is abundantly expressed and generates ROS in a calcium and PMA-dependent manner at rest (17). Moreover, NOX5 has a protective effect on rabbit arteries, and knocking out NOX5 will aggravate rabbit atherosclerosis (18). VSMCs sense mechanical stimuli conveyed by VECs, which continuously promote NOX production (19, 20). NOX-generated ROS activate many downstream molecules and channels, including MAPK, ERK, and AKT signaling, as a means of regulating downstream targets (21). The baculoviral inhibitor of the apoptosis repeat-containing protein 5 (BIRC5) gene (also known as survivin), a well-known target gene for cancer therapy, is involved in controlling multiple signaling pathways in tumor cells and has the dual function of inhibiting apoptosis and promoting cell proliferation (22). Although BIRC5 is closely associated with tumor development, it is not tumor-specific (22, 23). In previous studies, BIRC5 was found to be a key factor in the AKT pathway, which regulates the proliferation, migration, and survival of VSMCs in the arterial intima (9). Prevention of vascular occlusion triggered by excessive intimal proliferation in the grafted veins is an important therapeutic measure after revascularization. However, most previous studies only discussed the mechanisms of arterial IH with respect to the influence of hemodynamic-related factors, and few have examined the pathway-related factors affecting graft vein IH. Therefore, for graft vein IH, it is unclear whether OSS also regulates BIRC5 through oxidative stress to promote the proliferation, migration, and survival of VSMCs in vein grafts.

In this study, we used a rabbit external jugular vein graft model with increasing stenosis in the venous graft inflow tract artery. The flow rate was decreased to produce different levels of OSS and to study its association with graft vein IH. Our findings provide new ideas for the prevention of excessive IH in venous grafts and vascular remodeling.

## Materials and methods

### Experimental design

**Figure 1:**
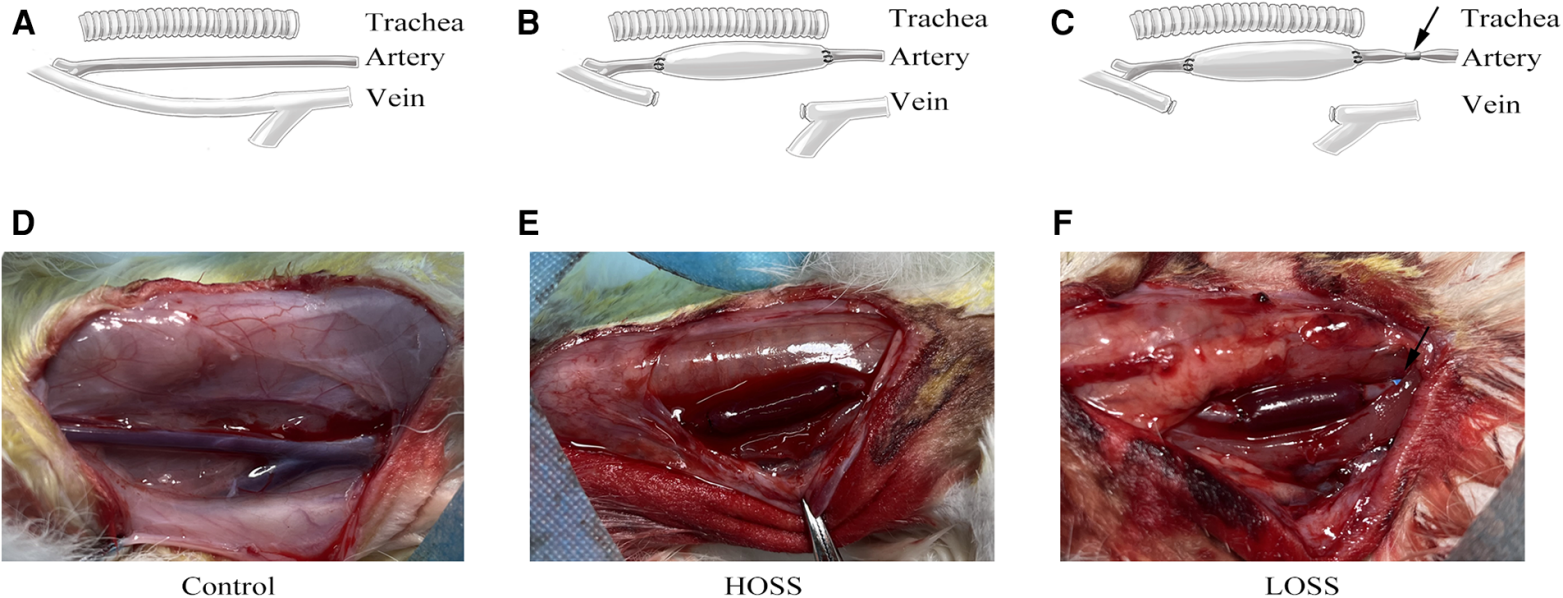
Surgical grouping and model construction. Rabbits were randomly divided into three groups: (**A,D**) control group (exposed vessels only), (**B,E**) high-oscillatory-shear-stress (OSS; HOSS) group (external jugular vein grafted to the ipsilateral common carotid artery), and (**C,F**) low-OSS (LOSS) group (vessels ligated to 50%–70% lumen area using a sheath at 5 mm of the inflow tract artery after vein grafting). *N* = 10 per group. Black arrow: 5F vascular sheath ligation.

Animals were provided by the Animal Center of Chongqing Medical University. The experiments were performed in accordance with the Animal Research: Reporting of *In Vivo* Experiments (ARRIVE) guidelines. Thirty male New Zealand rabbits (Yuda Experimental Rabbit Farm, Chongqing, China), weighing 2.0–2.5 kg, were randomly divided into three groups of 10 rabbits (Figure 1). Given the minimum reproducibility of the experiment, we considered this amount to be acceptable. In the control group, only the external jugular vein and common carotid artery were freed without grafting. In the high-OSS group (HOSS), the right external jugular vein was reversed and grafted to the ipsilateral common carotid artery. In the low-OSS group (LOSS), the inflow into the tract artery was restricted after receiving the venous graft.

### Surgical methodology

The rabbits were anesthetized by ear margin intravenous injection (0.5 ml/kg) and intraperitoneal injection (1 ml/kg) of 3% sodium pentobarbital. The animals' vital signs were closely observed during the operation, and if the rabbits developed temporary respiratory arrest owing to an excessive anesthetic dose, then mechanical ventilation was performed to assist respiration (Reward Life Sciences Co., Ltd., Shenzhen, China, cat. no. R407). Blood was then heparinized by intravenous injection of heparin solution (200 IU/kg) through the ear margins. After skin preparation, the surgical site was disinfected with iodophor disinfectant (5 g/l) for 5 min. Local anesthesia was administered to the surgical area by subcutaneous injection of 2.5 ml of 2% lidocaine. The skin was incised along the midline of the neck under aseptic conditions, and the right external jugular vein in the superficial fascia and ipsilateral paratracheal common carotid artery were completely separated using a non-contact approach. Non-invasive microscopic hemostatic clips were placed proximal and distal to the artery, the external jugular vein (2.0–3.0 cm) was harvested by ligating both ends of the vein, and a section of the common carotid artery (1.0–2.0 cm) was excised. The free vein was inverted after being flushed with heparin solution (6,000 IU/l), and end-to-end anastomosis of the free vein and artery was performed using an 8-0 non-invasive suture with an eight-stitch interrupted suture. After the sutures were completed and the hemostatic clips were opened to ensure that the graft vein was patent and there was no bleeding at either end, the suture incision was closed layer by layer. In the LOSS group, after successful grafting of the vein, an arterial sheath with appropriate shearing was placed in the arterial inflow tract, 5 mm proximal to the anastomosis. The arterial lumen diameter at this site was limited to 50%–70% of the normal lumen area by circumferential constriction sutures. Penicillin (800,000 IU) was administered intramuscularly for five days, and aspirin (50 mg) was administered orally for three days. New Zealand rabbits were placed in a 12 h light/dark cycle and fed with adequate food and water.

The procedure was repeated 4 weeks after surgery. After applying the same anesthesia, the original incision was reopened, the venous graft was cut. The harvested graft was approximately 2.5 cm in length. In order to ensure that the harvested graft would not be disturbed by the fused vessels at the suture, we selected the grafted vein 1−2 mm close to the suture and resected it. And the 4% paraformaldehyde-fixed tissue was embedded in paraffin for hematoxylin and eosin (H&E), Masson's trichrome (Masson), and immunohistochemical staining. Fresh tissue was used for immunofluorescence staining after frozen sectioning and western blotting. After the experiments, New Zealand rabbits were deeply anesthetized with a 3% pentobarbital aqueous solution with a high concentration of carbon dioxide ventricular rest, sacrificed, and the carcasses were placed in a special storage freezer in the animal center.

### Vascular ultrasonography

**Figure 2:**
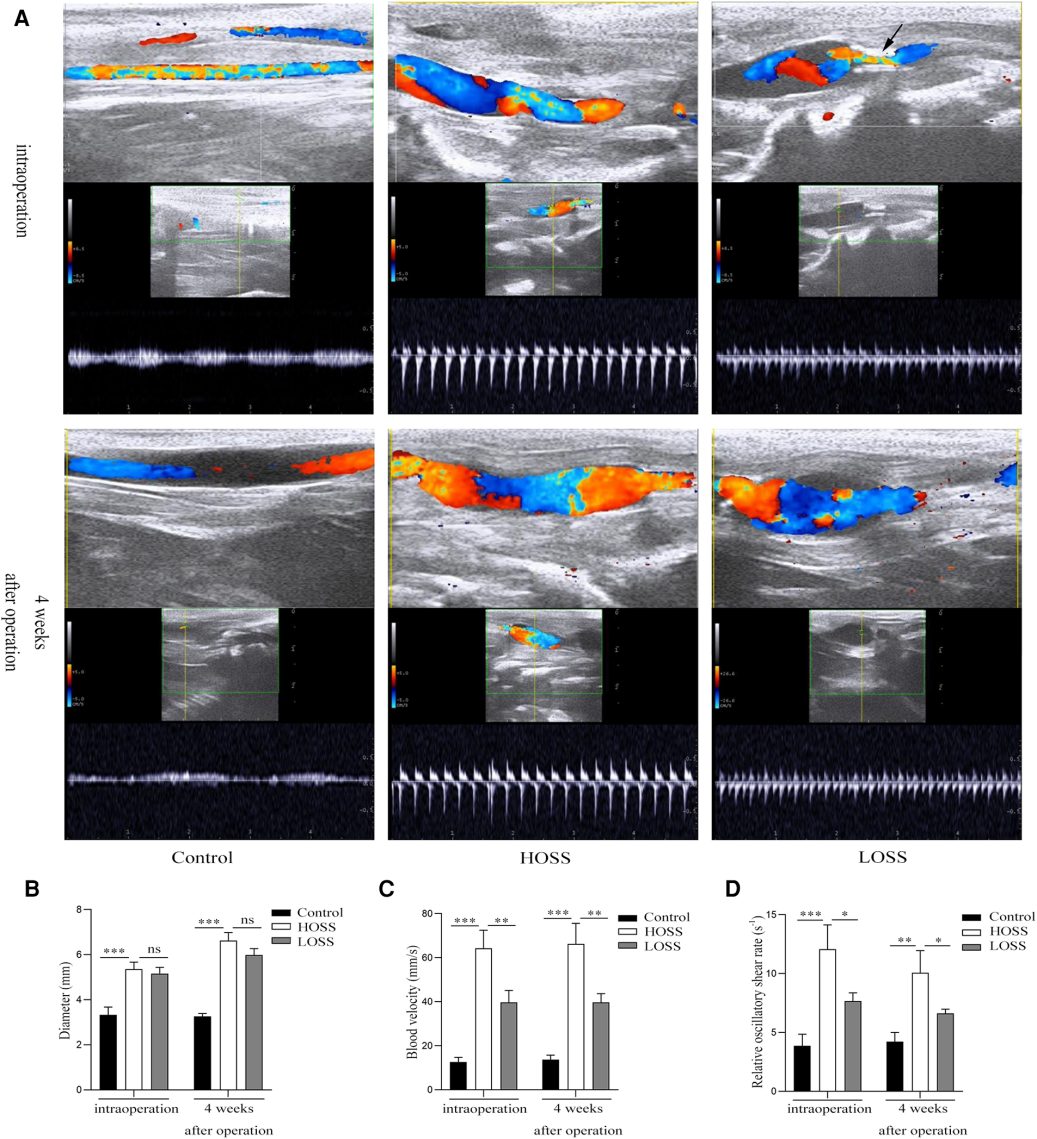
Vascular ultrasound shows changes of oscillatory shear stress (OSS) in grafted veins. (**A**) Vein graft morphology under ultrasound. (**B,C**) Columnar diagrams show the diameters and blood velocity. (**D**) Statistical results indicate that oscillatory shear rate differed among the three groups. Experiments were repeated in triplicate. *P < 0.05; **P < 0.01; ***P < 0.001; ns: no significant difference. Black arrow: inflow into the tract stenosis.

Successful venous graft model establishment was determined using vascular ultrasound (Figure 2). The blood flow velocity and vessel diameter of the venous grafts were measured both intraoperatively and 28 days postoperatively using an animal color ultrasound machine (Kyle Medical Instruments Ltd., cat. no. KR-S80). OSS was calculated according to the formula OSS = 8*η*v^mean^/d to determine the difference in OSS between the different experimental groups.

### Histological examination

Graft vein tissues were fixed in 4% paraformaldehyde for 24 h, embedded in paraffin wax, and cross-sectioned into 4 μm sections. Tissue sections were placed on an electric heating plate at 60–70 °C for 3–4 h, followed by dewaxing using xylene for 15 min at room temperature (20–25 °C). Dewaxing was completed after two repetitions. The sections were subsequently hydrated in a gradient of decreasing ethanol and washed with tap water for 5 min to remove excess ethanol. Sections were then H&E and Masson stained to examine the structural changes and degree of hyperplasia of the graft vein. The stained sections were observed under a light microscope (magnification, ×100; Leica, Wetzlar, Germany). The thickness of the intima and media was measured using ImageJ, and the intima/media ratio was calculated simultaneously. Three different parts of each sample were randomly selected for measurement, and the average value was calculated.

For H&E staining (Shanghai Biyuntian Biotechnology Co., Ltd., cat. no. C0105S), the sections were stained with hematoxylin for 4–5 min and washed with tap water for 5 min. The sections were placed in a hydrochloric acid-ethanol fast differentiation solution (Shanghai Biyuntian Biotechnology Co., Ltd., cat. no. C0163S) for 10 s and washed with water for 5 min. The hydrated tissue sections were again immersed in an eosin staining solution for 3 min. The sections were subsequently dehydrated using graded increments of ethanol and coverslips were fixed with neutral gum.

For Masson staining (Beijing Solarbio Technology Co., Ltd., cat. no. G1340), sections were stained with Ponceau S dye for 5 min and washed with tap water for 5 min. The sections were incubated with 1% phosphotungstic acid solution for 5 min and then stained with aniline blue dye for 5 min. Subsequently, they were incubated with 1% glacial acetic acid in water for 1 min, dehydrated with graded increments of ethanol, and fixed with neutral gum.

### Immunohistochemical staining

A mouse SP kit (Beijing Zhongsugi Jinqiao Biological Co., Ltd., cat. no. SP-9002) was used for antigen detection. The hydrated complete paraffin sections were restored using sodium citrate antigen repair solution and boiled for 20 min in a microwave oven. Subsequently, they were washed in phosphate-buffered saline (PBS) buffer for 5 min. This process was repeated three times. The sections were dried on filter paper and incubated in an appropriate amount of endogenous peroxidase blocking agent for 10 min at room temperature (20–25 °C). The sections were incubated with normal goat serum working solution for 30 min at room temperature. Sections were incubated overnight at 4 °C with primary antibodies against *α*-SMA (1:200; Proteintech, cat. no. 55135-1-AP), PCNA (1:200; Bioss Antibody, cat. no. bsm-2006M), MMP-2 (1:100; Bioss Antibody, cat. no. bs-20705R), and MMP-9 (1:100; Bioss Antibody, cat. no. bs-20705R). Afterward, the sections were incubated with a biotin-labeled goat anti-mouse IgG for 50 min, followed by dropwise incubation with horseradish enzyme-labeled streptavidin working solution for 15 min. The sections were stained with hematoxylin staining solution for 3 min after 30 s of incubation by adding freshly prepared DAB chromogenic solution (Beijing Zhongsugi Jinqiao Biological Co., Ltd., cat. no. ZLI-9018). The sections were dehydrated in graded increments of ethanol and fixed in neutral gum. Images were observed using a light microscope (magnification, ×200; Leica) and analyzed using ImageJ (version 1.8.0; National Institute of Health, Bethesda, MD, USA) software (https://imagej.nih.gov/ij/). The cumulative optical density value and area of each image were measured and used to calculate the average integrated optical density value at the site (IOD/area).

### Immunofluorescence staining

Briefly, freshly isolated graft veins were embedded in tissue frozen OCT compound (Sakura Fintek, CA, USA), cut into 4 *μ*m thick sections, and placed on slides. Dihydroethidium dissolved in dimethyl sulfoxide (40 *μ*mol/l) was added to tissue sections and incubated at 37 °C for 45 min in the dark (9). After washing three times with PBS, a blocker containing DAPI was added dropwise. Sections were placed under an orthomosaic fluorescence microscope (magnification,×100; Leica) and analyzed for fluorescence intensity using ImageJ.

### Western blotting

The isolated fresh tissues were ground using a tissue mill, and the tissue lysates were extracted from the tissues by adding the appropriate amount of lysis buffer (pH 7.4, RIPA:PMSF:phosphatase inhibitor = 100:1:2), followed by low-temperature high-speed centrifugation (4 °C, 12,000 *g*). The supernatant was aspirated and the bicinchoninic acid method was used to determine the protein concentration. Equal amounts of total protein from different samples were subjected to sodium dodecyl sulfate-polyacrylamide gel electrophoresis and transferred to nitrocellulose membranes. Then, 5% skim milk powder was used to block the nitrocellulose membranes for 90 min, and then they were washed with TBST buffer for 10 min. The membranes were then incubated with targeting NOX1 (1:1,000; Novus, cat. no. NBP1-31546), NOX2 (1:1,000; Proteintech, cat. no. 19013-1-AP), AKT (1:500; Wanlei Bio, cat. no. WL0003b), *p*-AKT (1:500; Wanlei Bio, cat. no. WL0003b), BIRC5 (1:500; Wanlei Bio, cat. no. WL03492), PCNA (1:2,000; Bioss Antibody, cat. no. bsm-2006M), BCL-2 (1:1,000; Abcam, cat. no. ab16904), BAX (1:500; Wanlei Bio, cat. no. WL01637), caspase-3/cleaved caspase-3 (1:500; Wanlei Bio, cat. no. WL02117), and *β*-actin (1:5,000; Bioss Antibody, cat. no. bsm-33036M) primary antibodies at 4 °C overnight (12–16 h). The membranes were washed three times with TBST buffer for 10 min each. After incubation with horseradish peroxidase-conjugated secondary antibody (1:2,000; Cell Signaling Technology, cat. no. 91196S) for 90 min at room temperature, the signal intensity was observed using chemiluminescence (Bio-Rad, CA, USA) according to the manufacturer's instructions. Image grayscale values were analyzed using ImageJ.

### Statistical analysis

All data are expressed as mean ± standard deviation of at least three independent experiments. One-way analysis of variance (t-test with least significant difference) was performed using SPSS (version 24.0; IBM, Armonk, NY, USA) to determine significant differences between the groups. Statistical significance was set at *P* < 0.05.

## Results

### Differences of OSS in grafted veins

All rabbits survived and both immediate and 28-day postoperative vascular ultrasound revealed the patency of venous grafts (Figure 2A). The diameters in the HOSS group were similar to those in the LOSS group both postoperatively and 28 days after surgery, and both groups had increased diameters relative to the control group (Figure 2B). Moreover, the diameter 28 days after surgery was significantly larger than that in the immediate period in both experimental groups (*P* < 0.05), and the change in diameter was the same in both groups postoperatively, whereas there was no significant change in the control group. The flow rate in the HOSS group was significantly greater than that in the LOSS group, and both were significantly greater than that in the control group (*P* < 0.05, Figure 2C). Contrary to the above results, there was no significant change in vascular flow velocity in any of the three groups in the immediate postoperative period and at 28 days. In addition, the shear rate (v/d) of the HOSS group was significantly higher than that of the LOSS group, and both were significantly higher than that of the control group (*P* < 0.05, Figure 2D). The difference in the OSS was derived from the formula OSS = 8*η*v^mean^/d, and the shear rate was proportional to OSS. Therefore, the postoperative OSS went from high to low in the HOSS, LOSS, and control groups, confirming the validity of the animal model.

### Wall remodeling characteristics in grafted veins

**Figure 3:**
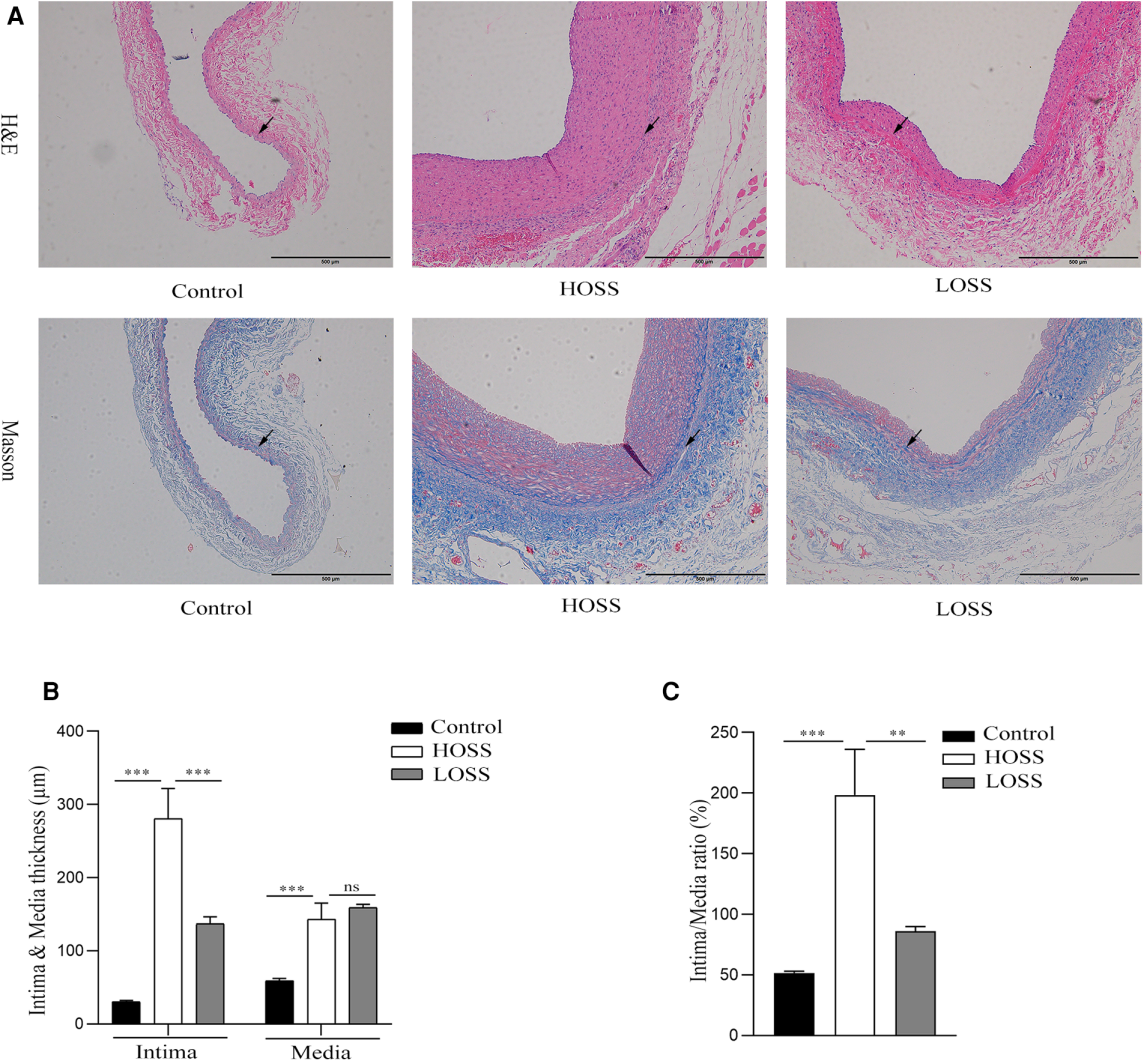
The extent of graft vein proliferation and structural changes. (**A,B**) Hematoxylin and Eosin (H&E) staining and Masson's Trichrome staining (magnification,×100). (**C,D**) Changes in intima and media thickness and intima/media ratio in the three groups. Visible deposition of collagen fibers (blue) in the graft vein to the media and muscle fibers (red) to the endothelium in Masson stain. Oscillatory shear stress (OSS) promoted vascular proliferation and accelerated graft vein remodeling. Experiments were repeated in triplicate. **P < 0.01; ***P < 0.001; ns: no significant difference. Black arrow: boundary between intima and media.

**Figure 4:**
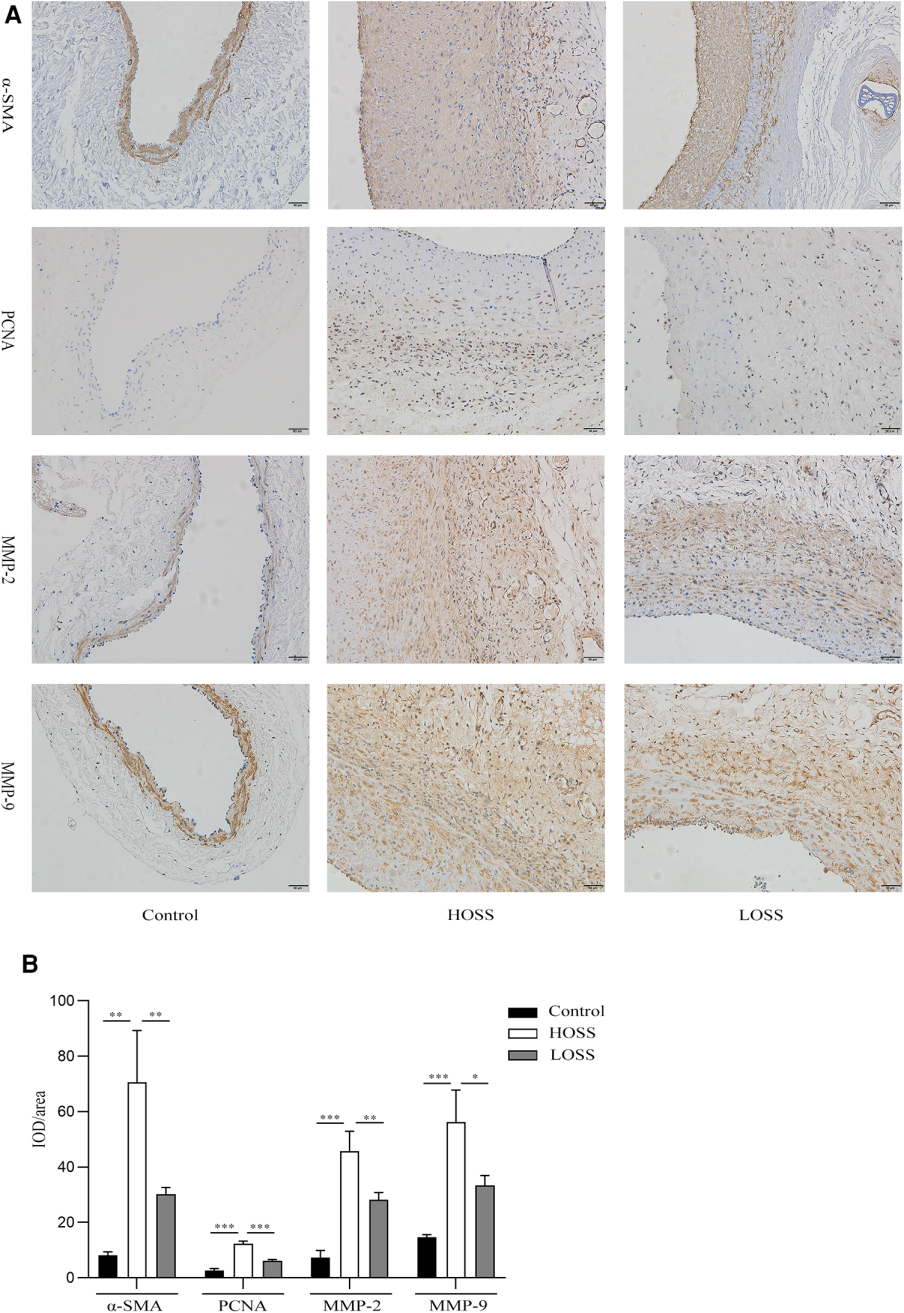
Effects of oscillatory shear stress (OSS) on proliferation and migration on vein grafts. (**A**) Immunohistochemical staining was performed to assess the expression of *α*-SMA, PCNA, MMP-2, and MMP-9 in vascular tissue (magnification,×200). (**B**) OSS promotes the expression of the above proteins, while its reduction slows down the proliferation and migration of vascular smooth muscle cells (VSMCs). Experiments were repeated in triplicate. *P < 0.05; **P < 0.01; ***P < 0.001.

Hyperplastic graft veins were isolated 28 days postoperatively and analyzed using H&E and Masson staining (Figures 3A,B). The intima and media were significantly thicker in the HOSS and LOSS groups than in the control group (*P* < 0.05, Figure 3C). Moreover, the intima/media ratio showed that the HOSS group was larger than the LOSS group, and both were larger than the control group (*P* < 0.05, Figure 3D). Masson staining showed that the main component of the intima was myofibrils, whereas collagen fibers were expressed more in the media. Compared to the control group, the HOSS and LOSS groups had relatively more disorganized collagen fibers. In addition, immunohistochemical staining was performed to observe protein expression sites and relative expression levels (Figure 4A). Statistical analyses (Figure 4B) revealed that OSS significantly induced the expression of *α*-SMA, PCNA, MMP-2, and MMP-9 in the hyperplastic intima and media of the venous grafts, and decreased OSS levels resulted in decreased expression (*P* < 0.05). We also found that PCNA, MMP-2, and MMP-9 were abundantly expressed in the media and adventitia membranes of grafted veins, while the expression was progressively diminished in the location near the sub-endothelium. These results suggested that the thickening of the grafted veins was due to OSS, which promoted the migration and proliferation of their subendothelial smooth muscle cells.

### OSS-induced changes in ROS

**Figure 5:**
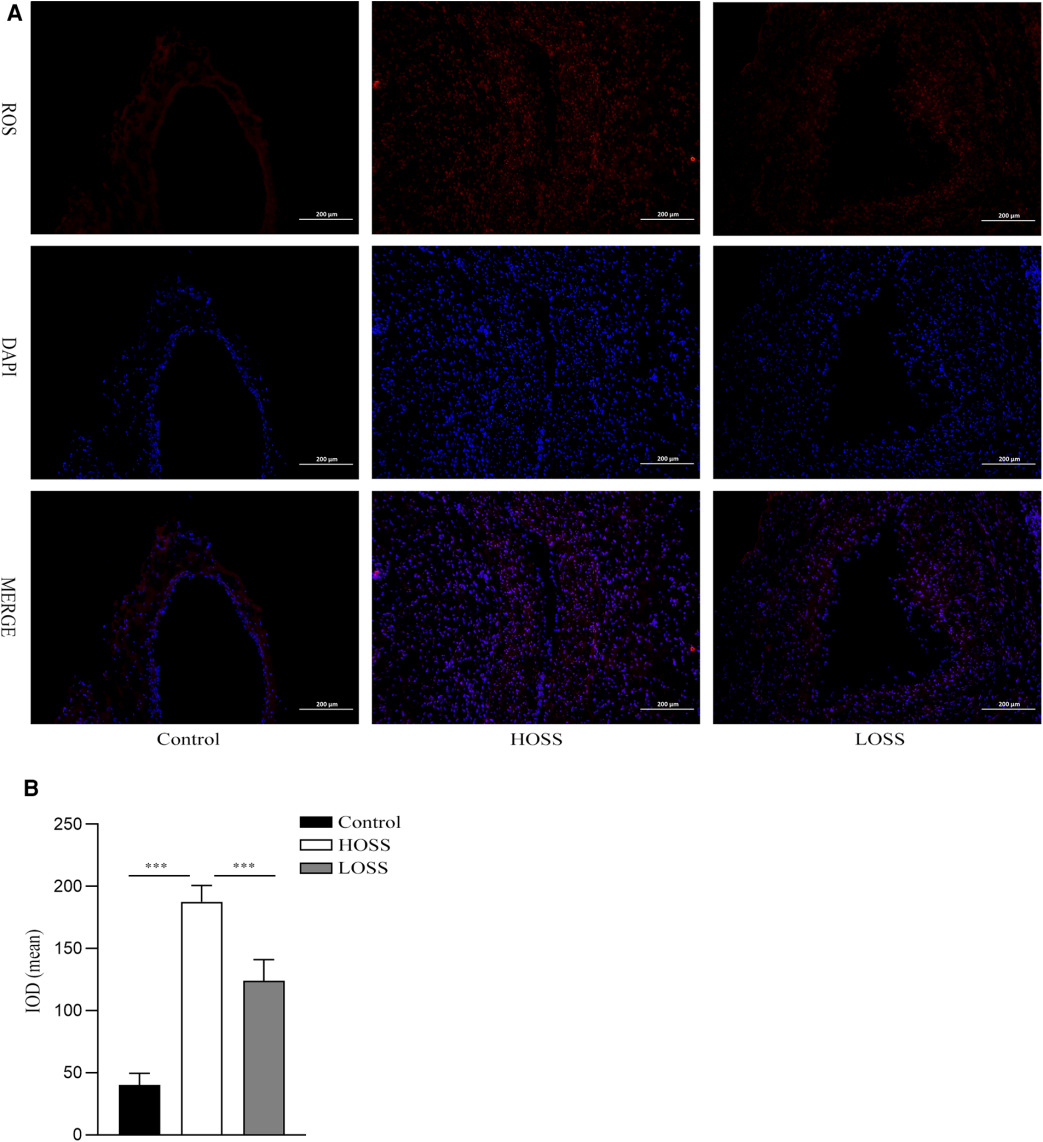
Oscillatory shear stress (OSS) upregulated reactive oxygen species (ROS) expression. (**A**) Immunofluorescence staining was used to measure the expression of ROS in the grafted veins. ROS (red) expression in grafted veins of different experimental groups was determined by immunofluorescence staining (magnification,×100). DAPI (blue) was used for nuclear staining. (**B**) Statistical analysis showed that the ROS fluorescence intensity was significantly increased in the high-OSS (HOSS) group and relatively diminished in the low-OSS (LOSS) group compared to that in the control group. Experiments were repeated in triplicate. ***P < 0.001.

Twenty-eight days after model establishment, ROS immunofluorescence staining was performed on the grafted veins to assess ROS levels in the different experimental groups (Figure 5A). The results showed that the basal ROS production in the control group was located in the subendothelial VSMC layer. ROS production was significantly increased in both grafted veins relative to that in the control group and was greater in the HOSS group than in the LOSS group (*P* < 0.05, Figure 5B); that is, high levels of OSS significantly promoted ROS expression through transduction of mechanical signals.

### OSS alters NOX-related signaling, cell proliferation, and apoptosis-related factor expression

**Figure 6:**
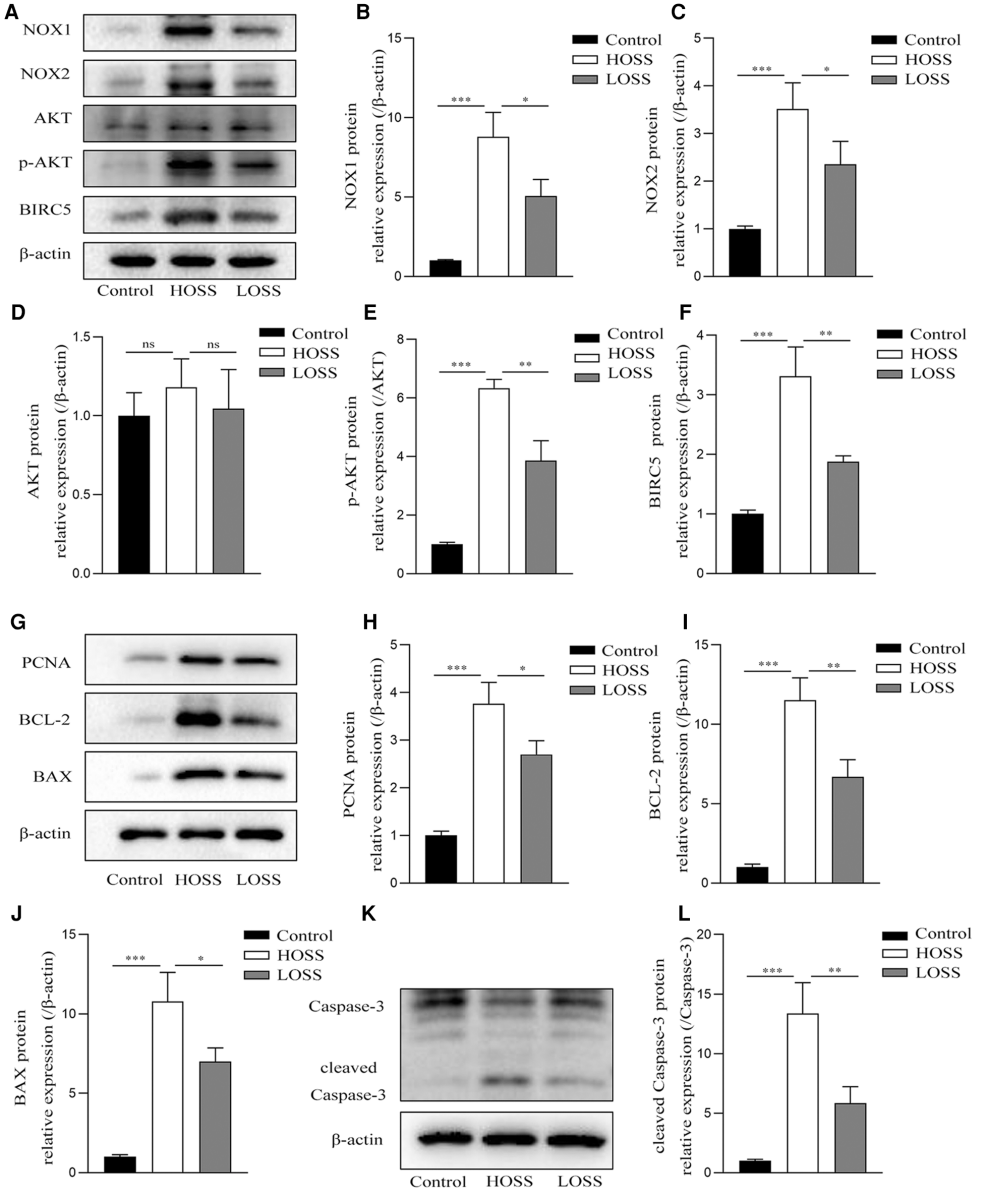
Oscillatory shear stress (OSS) is involved in altering NOX-AKT-BIRC5 signaling, cell proliferation, and apoptotic factor expression. Western blotting of purified protein lysates from grafted veins was used to detect protein expression. (**A–F**) OSS was positively correlated with the expression of NADPH oxidase-related proteins in grafted veins. (**G–L**) High OSS (HOSS) promoted the expression of proliferative, apoptotic, and anti-apoptotic related proteins, whereas decreasing the shear level attenuated their expression. Experiments were repeated in triplicate. *P < 0.05; **P < 0.01; ***P < 0.001; ns: no significant difference.

Next, protein lysates purified from grafted veins with different OSS were subjected to western blotting 4 weeks postoperatively (Figure 6). The expression of the pathway-related proteins NOX1, NOX2, *p*-AKT, and BIRC5 was significantly increased in the grafted veins of the HOSS group relative to that in the control veins, whereas reducing the OSS levels decreased the expression of several of these proteins (*P* < 0.05). That is, Alterations of ROS-producing NOX and ATK-BIRC5 in grafted vein VSMCs were associated with OSS, which promoted their proliferation, leading to IH. In addition, the expression trend of PCNA, a protein associated with cell proliferation, BCL-2, BAX, and cleaved caspase-3, a protein associated with apoptosis, was consistent with the above findings (*P* < 0.05). However, no statistically significant changes in total AKT protein expression were observed among the three groups.

## Discussion

The prevention of long-term patency decline due to excessive IH of the grafted vein has historically been the most important follow-up factor after autologous saphenous vein grafting (24, 25). Fluctuating OSS can significantly accelerate the development of IH through multiple signaling pathways, in which oxidative stress factors are implicated (10, 11). NOX-generated ROS activate MAPK, ERK, and AKT signaling, and BIRC5 is a key factor in the AKT pathway, which regulates the proliferation, migration, and increased survival of VSMCs in the arterial intima (21). To date, it remains unclear whether OSS regulates BIRC5 through oxidative stress to promote the proliferation, migration, and increased survival of VSMCs in vein grafts. Here, we sought to study the association of OSS with graft vein IH. To that aim, we used a rabbit external jugular vein graft model with increasing stenosis in the venous graft inflow tract artery, and the flow rate was decreased to produce different levels of OSS. Our results suggest that a certain degree of high OSS is an important factor causing IH and fibrosis in venous grafts. Arterial blood flow to the graft vein produced a higher OSS relative to the venous environment owing to mismatched diameters and fast flow rates, which was sufficient to induce oxidative stress in the graft vein. This was accompanied by an increase in ROS levels in the graft vein and the increased expression of ROS-producing NOX1 and NOX2 proteins and the downstream AKT-BIRC5, which ultimately lead to irreversible IH and tissue fibrosis.

Owing to their physiological structure, veins are much thinner than arteries (26). When transplanted into an arterial environment, several factors accelerate the remodeling of the grafted vein to “arterialize” to this rapidly changing environment, with OSS being one of the most important hemodynamic factors (25, 27–29). The graft vein undergoes both positive remodeling in the form of lumen enlargement and negative remodeling in the form of IH during the vascular remodeling process (3). Ultrasound results showed that significantly increased blood flow per unit time when grafted into the arterial circulation led to an early increase in the lumen diameter of the grafted vein. Rapid turbulent flow produces a higher OSS, causing injury and exfoliation of the endothelium, which induces vein graft reendothelialization. However, the nascent endothelium responds to the over-enhanced OSS by releasing various intracellular signaling molecules such as ROS, thus triggering the proliferation and migration of VSMCs and causing IH (30). *α*-Smooth muscle actin (*α*-SMA), a myofibroblast marker protein, is abundantly expressed in VSMCs. Masson and *α*-SMA immunohistochemical staining revealed the presence of a layer of annular endothelial VSMCs in normal veins, and *α*-SMA was expressed only in subendothelial SMC. In contrast, *α*-SMA was abundantly expressed in the intima and media layers in both grafted veins. Therefore, it is reasonable to speculate that the OSS in the arterial environment promotes the continuous proliferation of VSMCs in the grafted veins, while the proliferating SMCs migrate from the media to the intima, causing a large accumulation of subendothelial SMCs and resulting in IH.

Our results also confirmed that the expression of proteins associated with proliferation, survival, apoptosis, and migration (PCNA, BCL-2, BAX, caspase-3, MMP-2, and MMP-9) was increased in the grafted veins. In contrast, reducing OSS by narrowing the inflow tract diameter of the graft vein and slowing its flow velocity slowed the remodeling of the graft vein and decreased the expression of related proteins. This is consistent with the above reasoning that, although proliferation, increased survival, injury, and apoptosis are present simultaneously in the process of vein remodeling, a certain degree of high OSS in the arterial environment still accelerates the proliferation of VSMCs, leading to more severe IH after 4 weeks.

In the peripheral vascular system, ROS are mainly produced by NOX, which is a downstream mediator of the OSS-triggered cellular events (31). Among the NOX family, NOX2 was the first NADPH oxidase identified, whereas NOX1 was its first homolog (also called MOX1), and both were shown to be mainly expressed in VECs and SMCs (13, 32). Animal experiments have confirmed that NOX1 and NOX2 are involved in vascular growth and remodeling and play important roles in vascular diseases such as hypertension and atherosclerosis (14, 33, 34). ROS, as a central link in oxidative stress, are similar to second messenger signaling molecules that activate many redox-sensitive signaling pathways and are important signaling molecules that regulate the structural and functional status of blood vessels (12). ROS are produced by VECs, SMCs, and arterial outer membranes. Excessive ROS production is strongly associated with inflammatory responses, atherosclerosis, diabetes, hypertension, and tumorigenesis (35). In a normal venous environment, basal expression of ROS occurs mainly in the intimal layer of the vein. We found that OSS enhanced the production of ROS in the venous vessel wall of *in vivo* grafts, which was positively correlated with the expression of NOX1 and NOX2. Although ROS are not exclusively generated by NOX, ROS generation in this experiment was closely associated with the increased expression of NOX1 and NOX2.

In VSMC dysfunction, NOX-derived ROS seem to play a central role in coordinating different initiation-altering factors (hemodynamic factors, cytokines, and growth factors), signaling mediators (PKC, integrins, AKT, ERK1/2, and NF-*κ*B), and other regulatory systems (systemic and local renin-angiotensin system and reactive nitrogen species) (35). When the expression of NOX-derived ROS in and around cells increases, the downstream AKT-BIRC5 axis is activated and the proliferation of VSMCs is promoted (36). BIRC5, also known as the survivin gene, is a member of the apoptosis inhibitory protein family that has received attention because of its unique role in regulating the proliferation of VSMCs. BIRC5 is regulated by upstream AKT (37). When intracellular ROS increases, it activates downstream AKT and accelerates its phosphorylation, thus promoting an increase in the activated form of *p*-AKT, which in turn regulates BIRC5 protein expression. BIRC5 promotes graft vein IH and is mainly associated with the promotion of VSMC proliferation and the migration of the macrophage system to cells (38, 39). In our experiments, although there was no difference in total AKT protein expression, the trend of change in *p*-AKT and BIRC5 expression was consistent with that of ROS production, which ultimately led to the proliferation of VSMCs in grafted veins and increased the expression of anti-apoptotic and migratory proteins. Therefore, we hypothesized that when veins are transplanted into the arterial setting, it is possible that subendothelial VSMCs are influenced by OSS to promote increased intracellular NOX expression, which positively regulates downstream *p*-AKT/BIRC5, leading to irreversible IH in transplanted veins.

Although the results of our study confirm that OSS promotes IH of vein grafts in rabbits, more research is required for its application in clinical settings. Human grafted veins experience higher blood flow and stronger OSS than in animals. Due to their thicker media and adventitia, when compared with rabbits, localized IH is already present before transplantation, which would accelerate the failure of grafted veins (40). Studies have pointed out that transplanting venous external stents can effectively delay venous remodeling and prolong survival time (41). This may be related to improving the compliance of grafted veins and restoring laminar flow in the blood vessels. To a certain extent, higher laminar shear stress can inhibit the proliferation of VSMCs, thereby delaying IH (8, 42). However, OSS is a stress that varies based on the overall blood flow direction, considering a vector, which can be considered as a low laminar shear stress (7). Moreover, in a long-term follow-up (>1 year) of human saphenous vein grafts, some factors, such as inflammatory cell infiltration, will accelerate atherosclerosis occurrence (43). Reducing OSS and prolonging the survival time of vein grafts are challenges that need to be resolved urgently in clinical settings. Although we have provided novel insights on the effect of OSS on IH, the present study had some limitations that should be addressed. The NOX-related pathways that promote vein graft IH are discussed in this paper; however, whether these pathways are specifically dominated by NOX1 or NOX2 remains unclear. To overcome this limitation, in future studies, we will inhibit NOX1 and NOX2 based on the present study model to further observe potential mechanisms and improve our understanding.

## Conclusion

We found that OSS promotes the proliferation, migration, and survival of subendothelial VSMCs in grafted veins, thereby promoting IH. This may be related to the regulation of downstream *p*-AKT/BIRC5 levels *via* the increase in ROS generation by NADPH oxidase. Therefore, drugs inhibiting this pathway might be used to prolong graft vein survival time after vein grafting. Furthermore, the pathway may be required for the remodeling of vein to arterial circulation.

## Data Availability

The original contributions presented in the study are included in the article/Supplementary Material, further inquiries can be directed to the corresponding author/s.
